# The risk of long-term cardiometabolic disease in women with premature or early menopause: A systematic review and meta-analysis

**DOI:** 10.3389/fcvm.2023.1131251

**Published:** 2023-03-21

**Authors:** Jiajun Liu, Xueshan Jin, Wenbin Liu, Wanying Chen, Lan Wang, Ziyi Feng, Jieming Huang

**Affiliations:** ^1^ First Clinical Medical College, Guangzhou University of Chinese Medicine, Guangzhou, China; ^2^ School of Traditional Chinese Medicine, Jinan University, Guangzhou, China; ^3^ Second Clinical Medical College, Guangzhou University of Chinese Medicine, Guangzhou, China; ^4^ Department of Gynecology, The First Affiliated Hospital of Guangzhou University of Traditional Chinese Medicine, Guangzhou, China

**Keywords:** cardiometabolic disease, type 2 diabetes, hypertension, hyperlipidemia, coronary heart disease, stroke, meta-analysis, early menopause

## Abstract

**Background:**

Transition into menopause is associated with an increased risk of cardiovascular disease (CVD). However, it is unclear whether the association exists between premature menopause (defined as age at menopause 40 years) or early menopause (defined as age at menopause 40–45 years) and CVD or cardiovascular risk factors. The aim of this review was to comprehensively evaluate and meta-analyze the most reliable evidence about the relationship between menopausal age and the risk of long-term cardiometabolic disease.

**Methods:**

A comprehensive literature search of the PubMed, Web of Science, and Embase databases from inception to October 1, 2022, for titles and abstracts with a restriction to English language papers led to the discovery of the studies. Data are expressed as the Hazard Ratio (HR) with 95% confidence intervals (CI). The degree of heterogeneity was measured using the I-square (*I*^2^) index.

**Results:**

921,517 participants from 20 cohort studies published between 1998 and 2022 were considered. Compared to women with menopause at age >45 years, women with premature menopause (PM) or early menopause (EM) had a higher risks of type 2 diabetes (RR: 1.32, 95% CI: 1.08–1.62; RR: 1.11, 95% CI: 0.91–1.36, respectively), hyperlipidemia (RR: 1.21, 95% CI: 1.05–1.39; RR: 1.17, 95% CI: 1.02–1.33, respectively), coronary heart disease (RR: 1.52, 95% CI: 1.22–1.91; RR: 1.19, 95% CI: 1.07–1.32, respectively), stroke (RR: 1.27, 95% CI: 1.02–1.58; RR: 1.13, 95% CI: 0.97–1.32, respectively) and total cardiovascular event (RR: 1.36, 95% CI: 1.16–1.60; RR: 1.14, 95% CI: 0.97–1.35, respectively). No difference was found for hypertension in PM or EM women (RR: 0.98, 95% CI: 0.89–1.07; RR: 0.97, 95% CI: 0.91–1.04, respectively). Additionally, we also found that PM women, but not EM women, were linked with an increased risk of ischemic and hemorrhagic stroke. However, this is not in line with the conclusion that both PM and EM had a higher risk of total stroke.

**Conclusion:**

Women with PM or EM have a higher risk of developing long-term CVD, compared to women with menopause at age >45 years. Therefore, we recommend early lifestyle interventions (e.g., maintaining a healthy lifestyle) and medical treatments (e.g., timely initiation of menopausal hormone therapy) to decrease the risk of cardiometabolic disease in early or premature menopausal women.

**Systematic Review Registration:**

PROSPERO, identifier CRD42022378750

## Introduction

Menopause is chronically defined as the cessation of menstruation for 12 months as a result of follicular function loss (1). The average age of menopause has mostly been estimated to range from 49 to 52 years (2). However, about 5% of the female population enter menopause between the ages of 40 and 45, a condition termed “early” menopause. Approximately 1% of women enter menopause before the age of 40, a condition termed “premature” menopause (also named primary ovarian insufficiency if the amenorrhoea is spontaneous) (3, 4). According to WHO statistics, by 2030, 1.2 billion women globally will be in perimenopause or postmenopause, with 4.7 million women experiencing menopause each year (5).

Except for the impact of vasomotor symptoms on women's quality of life, transition to menopause is also linked with a higher risk of osteoporosis, dementia and CVD (6). Although considerable evidence suggests that early menopause is linked with the incidence of cardiovascular morbidity and mortality, the relationship between age at menopause and cardiovascular risk factors or CVD remains relatively poorly established (7). Some meta-analyses regarding PM and cardiovascular risk factors were mainly extracted from cross-sectional studies, comparing endpoints, such as type 2 diabetes (T2D), hyperlipidemia (HL) and hypertension (HT), between women with menopause at age >45 years. For example, a recent systematic review including prospective and cross-sectional studies reported the association between reproductive lifespan characteristics and cardiovascular risk factors. However, half of the studies were cross-sectional and could not deduce etiological relations (cause-and-effect) (8). In addition, current studies have reported the association between age at menopause and CVD, but these studies had inconsistent exposure variables (mixing premature and early menopause for analysis) or only examined the association between PM and CVD but not EM (9, 10). Women live roughly a third of their lives after menopause, and this time can extend further for earlier menopausal women. Under the circumstances, it is important to evaluate the relation between earlier menopause and the incidence of CVD (11).

The aim of this study, which examined all available evidence from cohort studies with long-term follow-up, was to assess whether PM or EM increases the risk of cardiovascular risk factors or CVD events compared to menopause at age >45 years.

## Methods

### Search strategy

This systematic review and meta-analysis was conducted following the Preferred Reporting Items for Systematic Reviews and Meta-Analyses (PRISMA) 2020 guidelines (12), and was prospectively registered on PROSPERO (registration number: CRD42022378750). The following PICO (Population, Intervention or exposure, Comparison, Outcome) elements were applied as inclusion criteria for this review: (a) Population: postmenopausal women; (b) Intervention: women with early or premature menopause; (c) Comparison group: women with menopause at age >45 years; (d) Outcome: HT, T2D, HL, and CVD events, such as coronary heart disease (CHD). The search included subject terms and text keywords for EM or PM, as well as cardiovascular endpoints. Subsequently, we searched three online databases: PubMed, Embase, and Web of Science, from inception to October 1, 2022. Retrieved articles were imported into NoteExpress, and duplicate articles were excluded. The complete search strategy is available in the Supplementary Table S1.

### Study selection

Cohort studies were included if they fulfilled the following criteria: (a) conducted in postmenopausal women (either naturally or surgically); (b) the primary outcome of interest was T2D, HT, HL, or CVD events. (c) reported the HR (RR or OR) and the corresponding 95% CI for outcomes associated with EM or PM vs. the reference category. Exclusion criteria were set as follows: (a) intervention studies, animal studies, or conference abstracts; (b) reviews or case reports; (c) follow-up for less than 3 years; (d) less than 10 subject cases. Moreover, if multiple similar articles were published in the same cohort, we selected only the one that contained the most complete information.

### Data extraction

Two researchers (Liu J and Liu W) conducted data extraction and quality assessment respectively, and consulted the third researcher (Huang J) to resolve disagreements. The extracted data included the author's name, publication date, region of study, year baseline, population number, age at enrollment, age at menopause, follow-up duration, menopause category, primary outcomes, MHT/OC used, and multivariate-adjusted effect estimates (e.g., HR, RR, or OR) with corresponding 95%CI. The multivariate-adjusted effect estimate was used for further analysis in studies that reported both adjusted and multivariate-adjusted effect estimates.

### Study quality assessment

The Newcastle-Ottawa Scale (NOS) for quality evaluation of systematic reviews was applied to long-term follow-up cohort studies (13). The scale consisted of eight items divided into three sections: participant selection, cohort comparability, and outcome assessment. The total score was 9. Summary scores of ≤4, 4–7, and ≥7 represent low, moderate, and high quality studies, respectively, and all included studies scored more than 7 points. Detailed scores for each study can be obtained in Supplementary Table S2.

### Statistical analysis

We identified reference category in each article, and the majority had menopause ages of 50 to 54 years. Multivariate-adjusted HR or RR and 95% CI were extracted from the selected literature to measure the association between PM or EM and cardiovascular outcomes. The original HRs extracted from the articles were considered equivalent effects to RRs (14). *I*^2^ statistic was used to assess the heterogeneity between studies, with ≤50% representing no heterogeneity, >50% representing moderate heterogeneity, and >70% representing high heterogeneity. When there was no heterogeneity between studies, we chose a fixed-effects model to conduct the analysis, if not, the random-effects model was selected. The random-effects model was used to conduct meta-analysis of outcomes (T2D, HT, CHD, and stroke) since the included literatures might differ statistically and clinically to some extent. When there existed high heterogeneity (*I*^2^ > 70%), sensitivity analysis were conducted excluding literatures with a large heterogeneity (outliers) (15). Furthermore, Egger's test was applied to assess potential publication bias. All analyses were performed with Stata software, version 15.1 (Stata Corp LP, Texas, United States).

## Results

### Literature search

**Figure 1:**
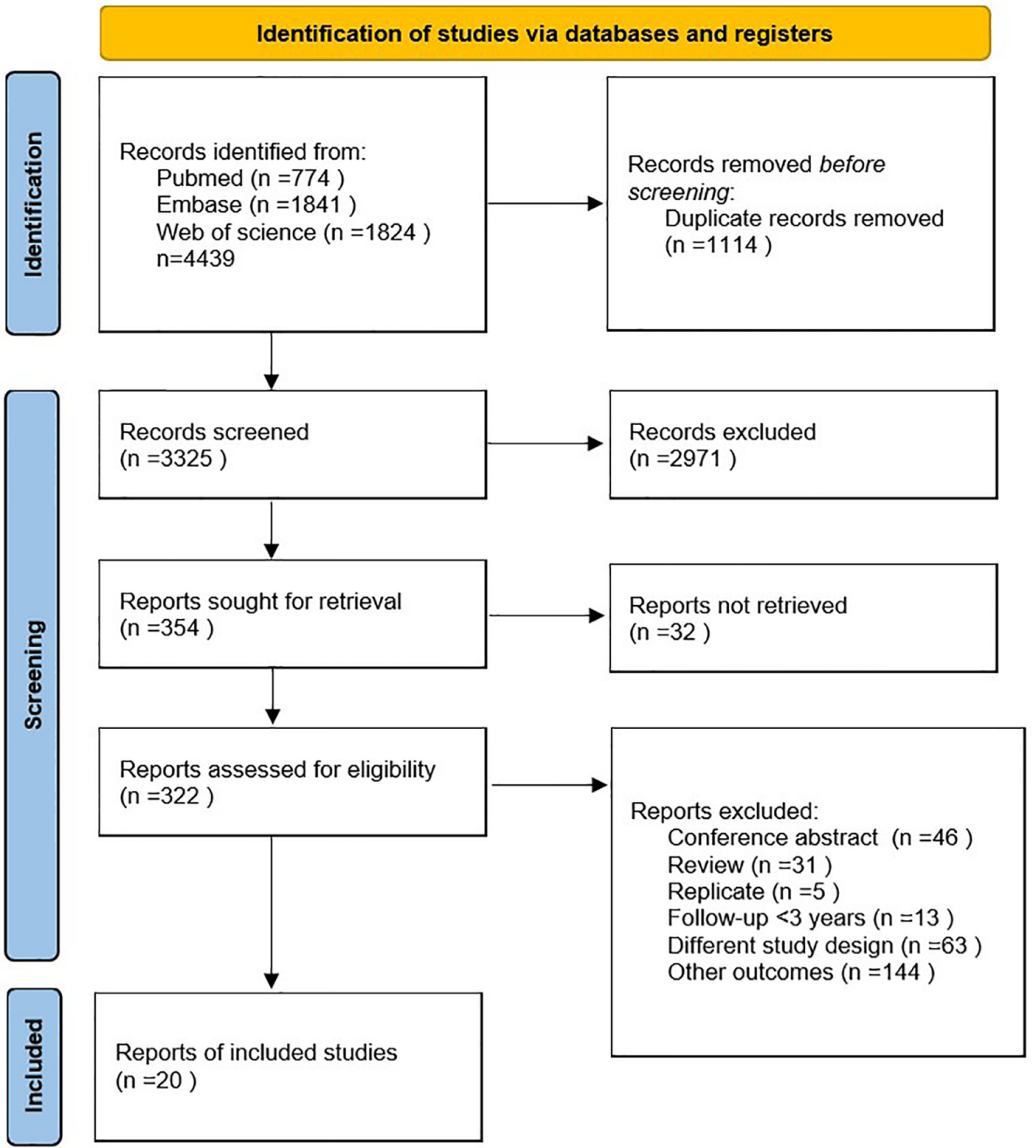
Flowchart for searching and selection details.

A total of 4,439 articles, including 1,114 duplicates, were retrieved from the three databases. After reading the titles and abstracts of the 3,325 articles, 2,971 were excluded because they were not relevant to the study. Of the remaining 354 studies, 334 were excluded due to conference abstracts (*n* = 46), reviews (*n* = 31), replicates (*n* = 5), follow-up < 3 years (*n* = 13), different study designs (*n* = 63), other outcomes (*n* = 144), and reports not retrieved (*n* = 32). Finally, 20 cohort studies were included (4, 16–34). The detailed literature on inclusion or exclusion can be obtained from Figure 1.

### Study characteristics

Among the 20 articles, publication years ranged from 1998 to 2022, consisting of 921,517 patients. With respect to the regions, six were conducted in Asia (17, 20, 21, 29–31), six in Europe (18, 24, 26–28, 34), five in North America (4, 22, 25, 32, 33), two in South America (19, 23) and one was a pooled study (16). The follow-up duration ranged from 4 to 67 years, and the age at enrollment varied from 26 to 76 years. All articles used self-reported menopausal ages. Ascertainment of cardiovascular risk factors or CVD events was determined according to medical records, official certificates, International Classification of Diseases (ICD) codes, or self-reporting (such as hypertension and hyperlipidemia). Most studies adjusted for smoking as a confounder, except for these six studies (20, 21, 24, 25, 31, 33). In five studies (18, 25, 27, 31, 32) women who used or had used menopausal hormone therapy (MHT) were excluded from analysis, and in one study (24) women did not use MHT. In five studies (17, 19–21, 34) MHT use was not available. In five studies (4, 16, 22, 23, 29) it was used as a confounding factor and in four studies (26, 28, 30, 33) women who used MHT/OC and not used were analyzed separately. Most of the studies only included women in natural menopause (4, 16, 19, 22, 24, 25, 28–33), four studies separately analyzed the impact of surgical and natural menopause on cardiovascular outcomes (17, 20, 26, 34), two studies did not distinguish between natural and surgical menopause (18, 27), and one study had unknown the type of menopause (21). The baseline characteristics included in this analysis are shown in Table 1.

**Table 1:** Characteristics of the included cohort studies.

Study	Cohort	Region of study	Year baseline	Population number	Age at enrollment (mean years SD or range)	Follow-up Duration (mean years SD or range)	Menopause category	Primary outcome	MHT used	Adjusted factors
Baba 2010	Jichi Medical School Cohort Study	Japan	1992–1995	3868	61.0 ± 0.7	10.8 ± 2	<40, 40–44, 45–49, 50–54^r^, ≥55	Stroke	Not available	Age, blood pressure, total cholesterol, BMI, smoking and alcohol drinking habits
Blumel 2022	Chilean Women Cohort	Chile	1990–1993	1119	47	Median29.2	<40, >40^r^	T2D, HT	Not available	Age, LDL-C, HDL–C, TG, number of cigarettes per week
Brand 2013	InterAct Study	Europe	1991–2000	7864	59.2 ± 5.8	Median10.7	<40, 40–44, 45–49, 50–54^r^, ≥55	T2D, HT, HL	Excluded	Age at entry, diabetes risk factors, reproductive factors, smoking
Choi 2005	Korean Elderly Pharmaco epidemio- logic Cohort	Korea	1993–1998	5731	≥65 69.8 ± 5.5	6	<40, 40–44, 45–49, 50–54^r^, ≥55	Stroke, HT	Excluded	Age at enrollment, physical activity, a history of hypertension
Cooper 1998	National Health and Examination Survey I	USA	1971–1975	2562	50–74	4	<40, 40–44, 45–49, ≥50^r^	CHD; stroke	Confounder	Age, duration of follow-up, race, education, smoking, and use of hormone replacement therapy
Gallagher 2011	Shanghai Textile workers	China	1989–1991	98,180	30->60	10	<40, 40–44, 45–49, 50–54^r^, ≥55	CHD	Not available	Age
Hong 2007	Kangwha Cohort	Korea	1985	2658	66.0 ± 8.2	15.8	<40, 40–44, 45–49^r^, >50	Total cardiovascular events; CHD; stroke	Not available	Age, alcohol consumption, education, age at first birth, health level, marital partner, parity, age at menarche, oral contraceptive use, HT
Hu 1999	Nurse’ Health Study	USA	1976–1994	35,616	30–55	0–18	<40, 40–44, 45–49, 50–54^r^, ≥55	CHD, Stroke	Excluded	Age; BMI; history of myocardial infraction,, hypercholesterolemia, diabetes; smoking; parity
Jacobsen 1999	California Seventh-Day Adventists	USA	1976	5279	≥26 49.2 ± 4.5	0–12	35–40, 41–44, 45–48, 49–51^r^, 52–55, 56–60	CHD	Separate analysis	Diabetes, HT, parity, age at first birth, physical activity in leisure
Jacobsen 2004	3 Norwegian County Study	Norway	1961	19,731	32–74	37	<40, 41–43, 44–46, 47–49, 50–52^r^, 53–55, 56–60	Stroke	Not used	Age, county, occupational group, birth cohort
Lay 2018	Health, Well-Being and Aging Study	Brazil	2000	1265	Median70	16	≤40, 41–44, 45–49, 50 54^r^, ≥55	Total cardiovascular events	Confounder	Birth year, education, marital status, race, parity, smoking, number of chronic diseases and estrogen therapy
Li 2013	Black Women's Health Study	USA	1995	11,212	21–69	13	<40, 40–44, 45–49, 50–54^r^, ≥55	Total cardiovascular events	Confounder	Age, time, period, education, maritalstatus, BMI, smoking, alcohol consumption, physical activity, dietary pattern, reproductive factors, age at menarche and firrst birth, parity, oral contraceptive use, lactation duration, unilateral oophorectomy
Løkkegaard 2006	Danish Nurse Cohort Study	Denmark	1993	7102	>44	0–6	<40, 40–45, >45^r^	CHD, HT	Separate analysis	Smoking, self-rated health, use of antihypertensive drugs, BMI, presence of HT, angina, diabetes
Mondul 2005	Cancer Prevention Study II	USA	1982	68,154	57	20	40–44, 45–49, 50–54^r^	CHD; stroke	Excluded	Age, race, marital status, BMI, age at menarche, parity, education, alcohol consumption, oral contraceptive use, exercise
Muka 2017	Rotterdam Study (RSI, RSII, RSIII)	Netherlands	1990–1993; 2000–2001; 2003	3639	66.9 ± 9.6	Median9.2	<40, 40–44, 45–55, >55^r^	T2D	Separate analysis	Age, RSI, RSII and RSIII, hormone replacement therapy, age at menarche, BMI, glucose, insulin, TC, lipid-lowering medication, systolic BP, antihypertensive medications, alcohol intake, smoking, education, prevalent CVD, physical activity, CRP
Ossewaarde 2005	Diagnostisch Onderzoek Mammacarcinoom Cohort	Netherlands	1974–1977	12,134	48–68	17 ± 5.1	<40, 41–44, 45–50, ≥50^r^	Total cardiovascular events; CHD; stroke	Excluded	Age, type of menopause, oral contraceptive use, parity, BMI, smoking, HT, diabetes mellitus, previous CVD
Wang 2022	China Kadoorie Biobank Study	China	2004–2008	281,319	50.9 ± 10.4	10.8	<40, 40–44, 45–49^r^, 50–53, ≥54	T2D	Confounder	Education, household income, smoking, alcohol drinking, physical activity, anthropometric measurements, health status of HT, family history of diabetes, reproductive factors, oral contraceptive
Welten 2021	EPIC-NL Cohort	Netherlands	1993–1997	16,244	58.2	15	<40, 40–44, 45–49, 50–54^r^, >55	Stroke; HL	Not available	Age at enrollment, smoking, systolic blood pressure, BMI
Wu 2014	Shanghai Women's Health Study	China	1996–2000	36,402	40–70	11.2	<40, >40^r^	Total cardiovascular events, CHD, Stroke, T2D	Separate analysis	Age at enrollment and menarche, occupation, income, current smoking, type of menopause, nulliparity, hormone replacement therapy
Zhu 2019	InterLACE	Australia, Scandinavia, United States, Japan, UK	1946–2013	301,438	57.0 ± 10.3	0–67	<40, 40–44, 45–49, 50–51^r^, 52–54, ≥55	CHD; stroke	Confounder	Age at last follow-up, ethnicity, education level, BMI, smoking, HT, hormone therapy, oral contraception use, perimenopause group

T2D, type 2 diabetes mellitus; HT, hypertension; LDL-C, low-density lipoprotein cholesterol; HDL-C, high-density lipoproteincholesterol; HL, hyperlipidaemia; CHD, coronary heart disease; IHD, ischemic heart disease; BMI, Body Mass Index; CRP, C-reactive protein.

InterAct Study, a prospective case-cohort study nested within the European Prospective Investigation into Cancer and Nutrition; EPIC-NL Cohort, European Prospective Investigation into Cancer and Nutrition–Netherlands; InterLACE, The International Collaboration for a Life Course Approach to Reproductive Health and Chronic Disease Events.

r, reference catagory.

### Meta-Analysis outcomes

#### Cardiovascular risk factors

##### Type 2 diabetes

**Figure 2:**
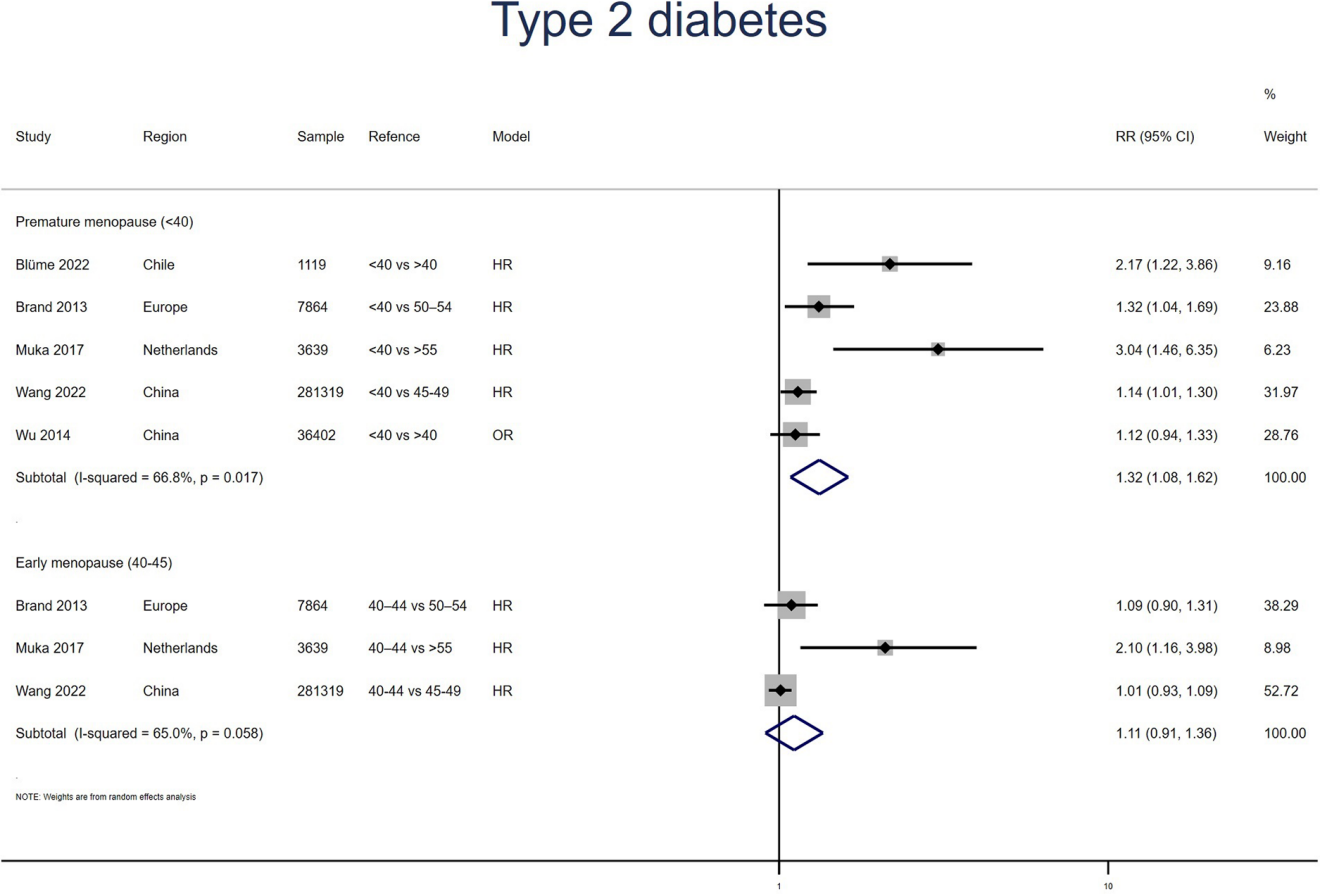
Forest plot presents the association between PM or EM and the risk of T2D in cohort study.

Five studies for PM and three studies for EM reported T2D. Meta-analysis showed that both PM (pooled RR: 1.32, 95% CI: 1.08–1.62, *I*^2^ = 67%) and EM (pooled RR: 1.11, 95% CI: 0.91–1.36, *I*^2^ = 66%) had an increased risk of T2D (Figure 2). No significant publication bias was found in Egger's test (*p* = 0.149 for PM, *p* = 0.161 for EM).

##### Hypertension

**Figure 3:**
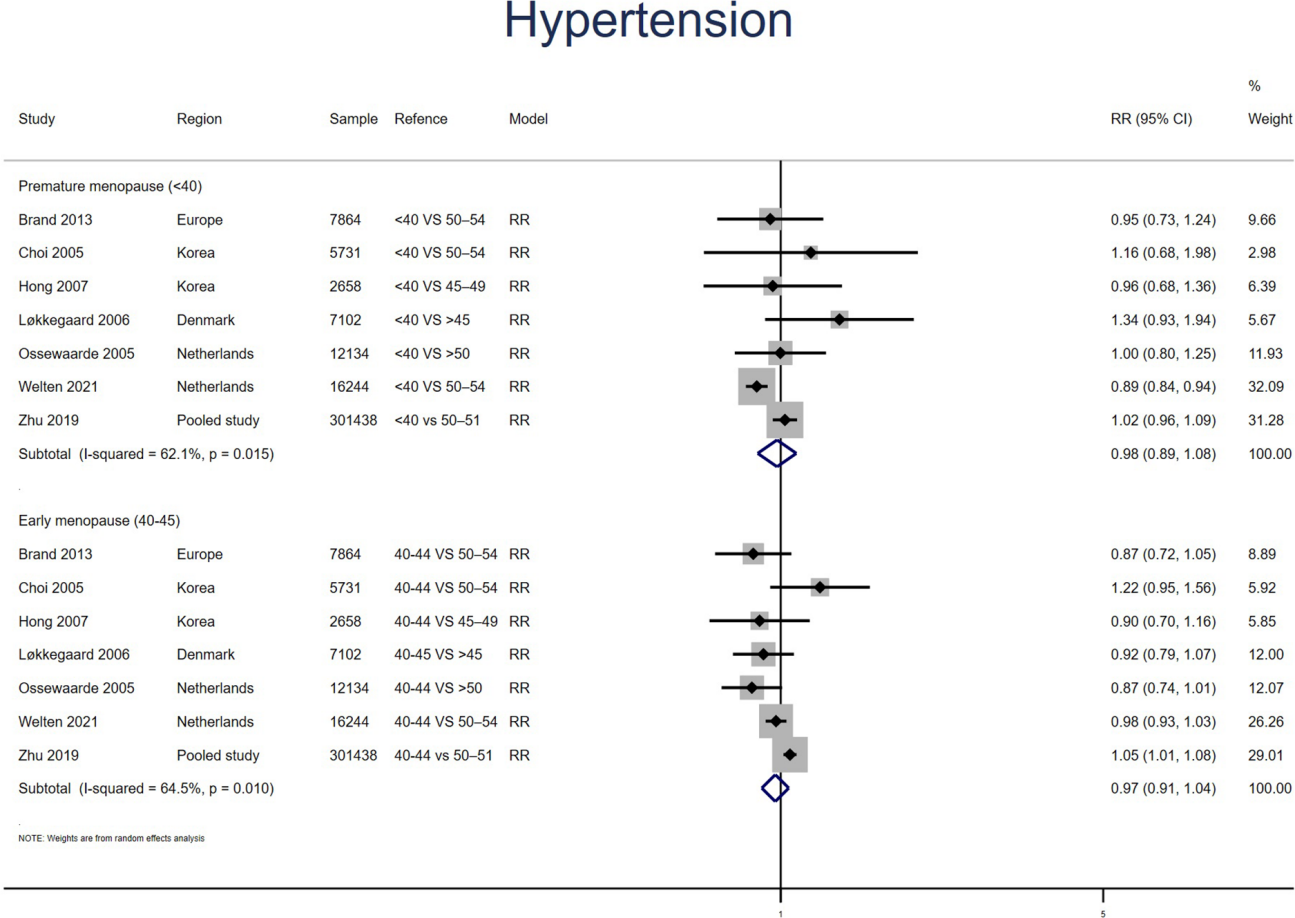
Forest plot presents the association between PM or EM and the risk of hypertension in cohort study.

Eight studies for PM and seven studies for EM reported HT. No significant effect was found for PM (RR: 0.98, 95% CI: 0.89–1.07, *I*^2^ = 56%) or EM (RR: 0.97, 95% CI: 0.91–1.04, *I*^2^ = 65%) in the meta-analysis of the estimations for risk of HT (Figure 3). No significant publication bias was found in Egger's test (*p* = 0.395 for PM, *p* = 0.179 for EM).

##### Hyperlipidemia

**Figure 4:**
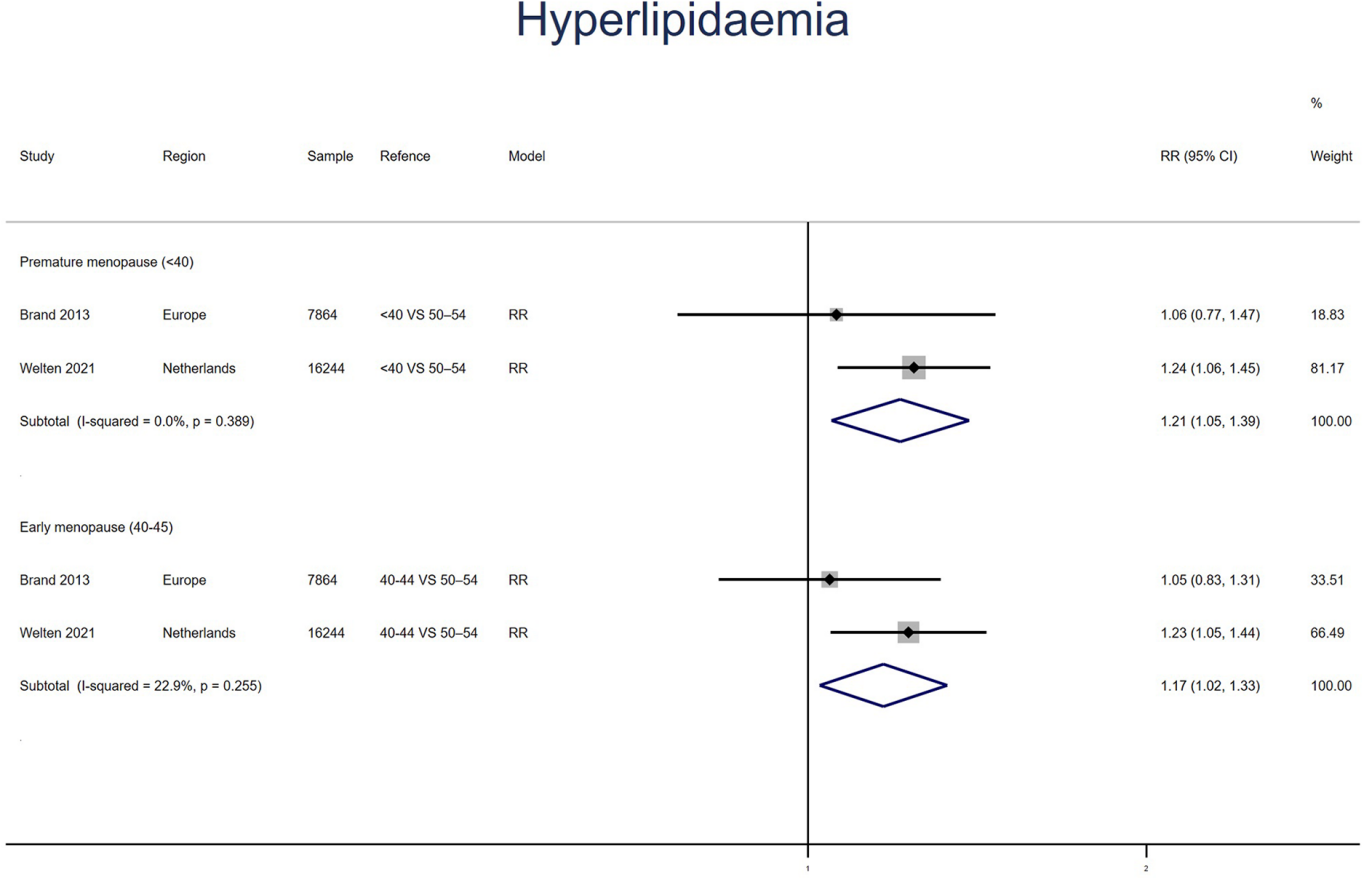
Forest plot presents the association between PM or EM and the risk of hyperlipidaemia in cohort study.

Two studies for PM and EM reported HL, with no heterogeneity between the studies (*I*^2^ = 0% for PM women, *I*^2^ = 23% for EM). Meta-analysis showed that both PM (pooled RR: 1.21, 95% CI: 1.05–1.39) and EM (pooled RR: 1.17, 95% CI: 1.02–1.33) had an increased risk of HL (Figure 4).

#### Cardiovascular disease events

##### Coronary heart disease

**Figure 5:**
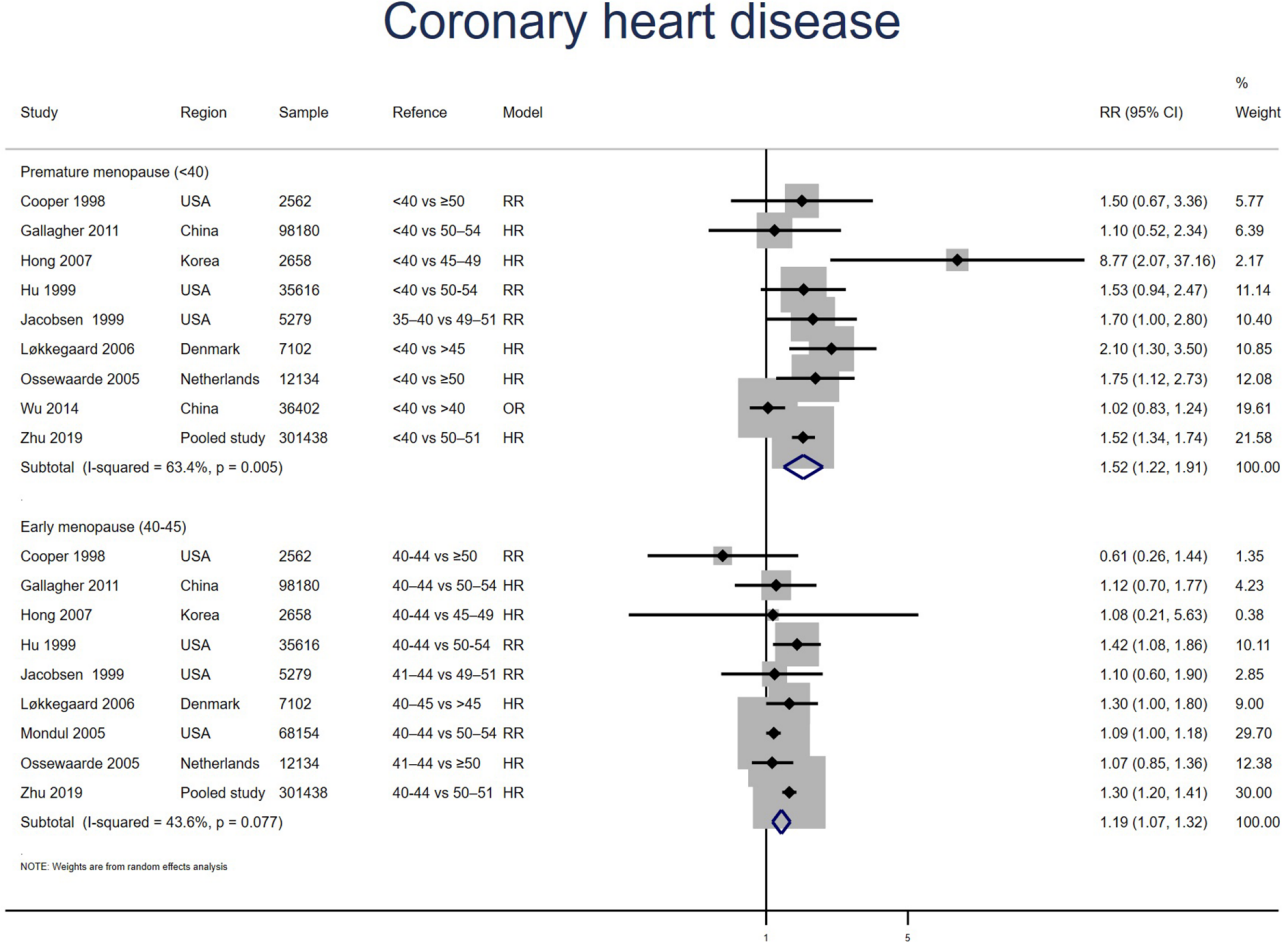
Forest plot presents the association between PM or EM and the risk of coronary heart disease in cohort study.

Nine studies for PM and EM reported CHD. Meta-analysis showed that both PM (pooled RR: 1.52, 95% CI: 1.22–1.91, *I*^2^ = 63%) and EM (pooled RR: 1.19, 95% CI: 1.07–1.32, *I*^2^ = 44%) had an increased risk of CHD (Figure 5). No significant publication bias was found in Egger's test (*p* = 0.323 for PM, *p* = 0.660 for EM).

##### Stroke

**Figure 6:**
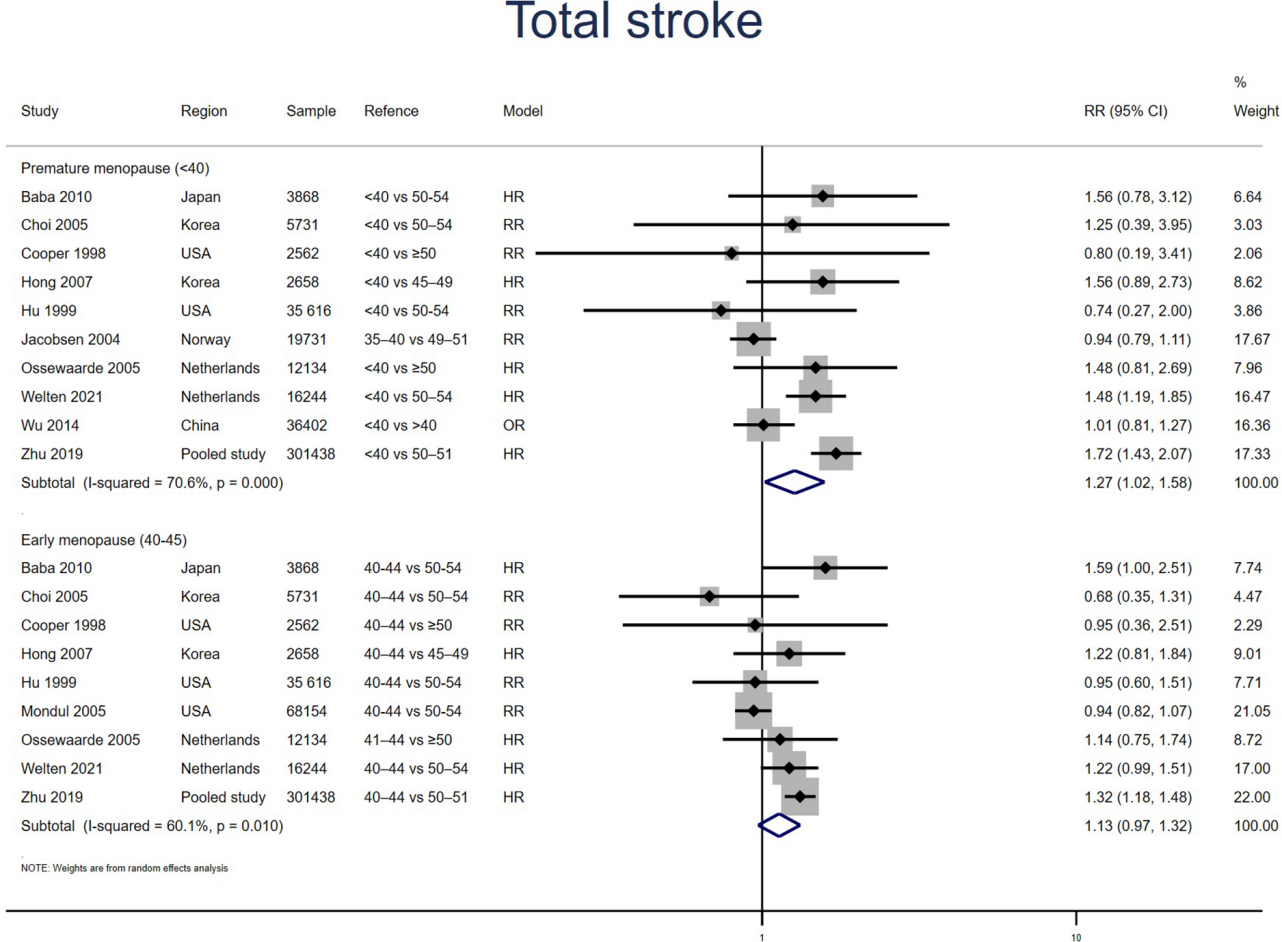
Forest plot presents the association between PM or EM and the risk of stroke in cohort study.

Ten studies for PM and nine studies for EM reported stroke. Meta-analysis showed that both PM (pooled RR: 1.27, 95% CI: 1.02–1.58, *I*^2^ = 71%) and EM (pooled RR: 1.13, 95% CI: 0.97–1.32, *I*^2^ = 60%) had an increased risk of stroke (Figure 6). Excluding two outliers in the PM group (16, 24) reduced the *I*^2^ statistic from 71% to 17% and produced a point estimate with a comparable range (pooled RR: 1.26, 95% CI: 1.06–1.51). No significant publication bias was found in Egger's test (*p* = 0.948 for PM, *p* = 0.683 for EM).

##### Ischemic stroke

**Figure 7:**
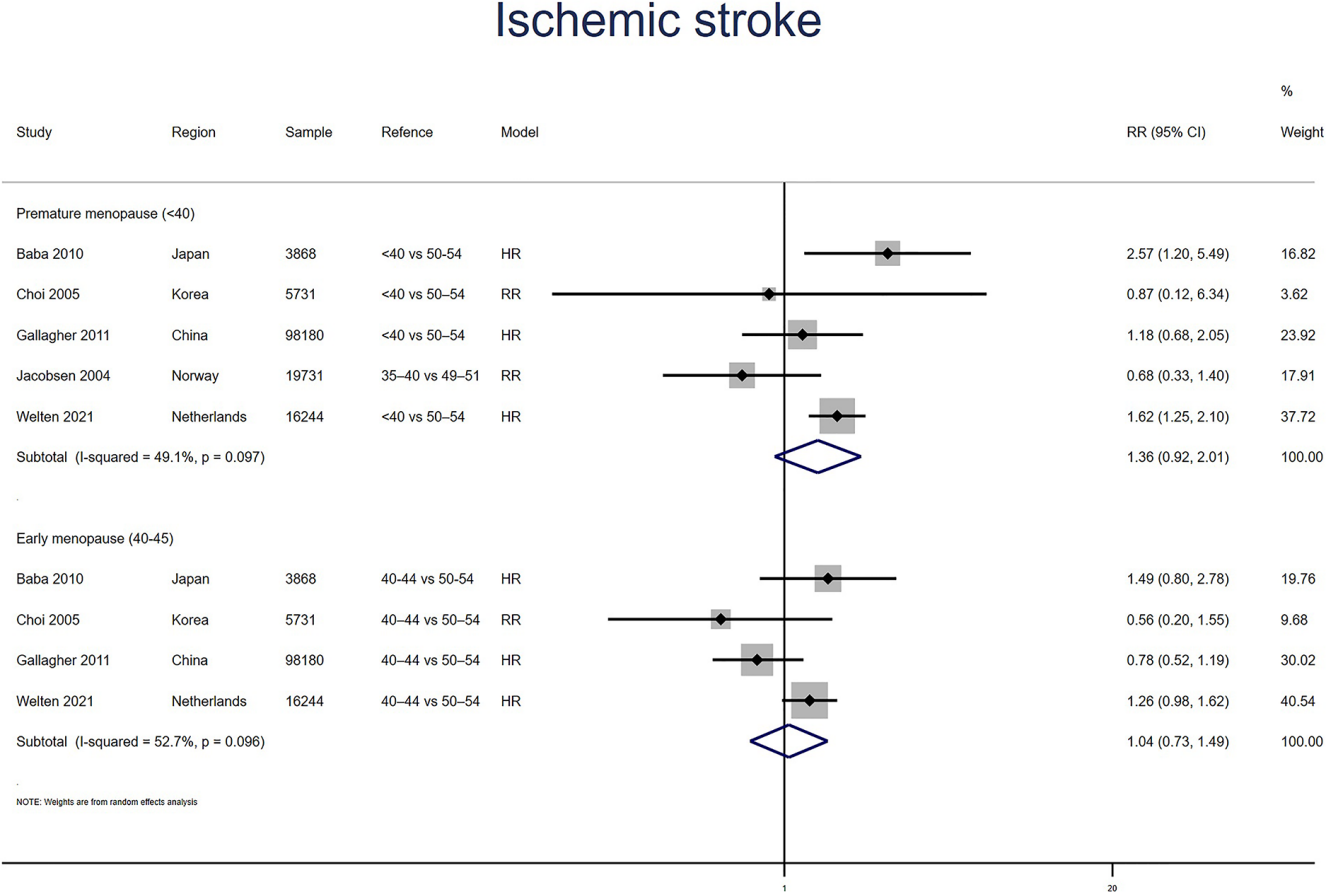
Forest plot presents the association between PM or EM and the risk of ischemic stroke in cohort study.

Five studies for PM and four studies for EM reported ischemic stroke. Meta-analysis showed that PM (pooled RR: 1.36, 95% CI: 0.92–2.01, *I*^2^ = 49%) but not EM (pooled RR: 1.04, 95% CI: 0.73–1.49, *I*^2^ = 53%) had an increased risk of ischemic stroke (Figure 7). No significant publication bias was found in Egger's test (*p* = 0.527 for PM, *p* = 0.511 for EM).

##### Hemorrhagic stroke

**Figure 8:**
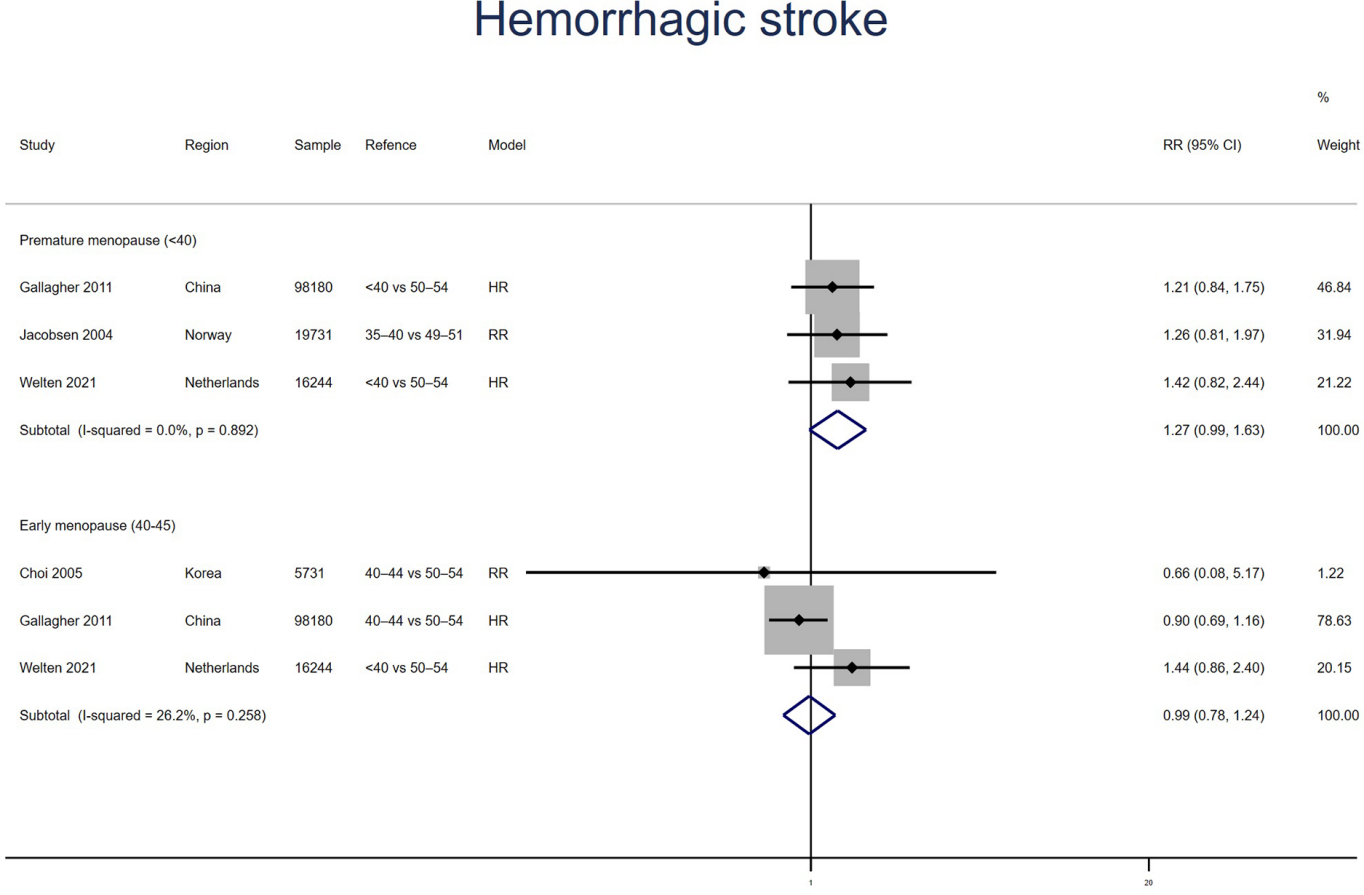
Forest plot presents the association between PM or EM and the risk of hemorrhagic stroke in cohort study.

Three studies for PM and EM reported hemorrhagic stroke. Meta-analysis showed that PM (pooled RR: 1.27, 95% CI: 0.99–1.63, *I*^2^ = 0%) but not EM (pooled RR: 0.99, 95% CI: 0.78–1.24, *I*^2^ = 26%) had an increased risk of hemorrhagic stroke (Figure 8). No significant publication bias was found in Egger's test (*p* = 0.140 for PM, *p* = 0.842 for EM).

##### Total cardiovascular events

**Figure 9:**
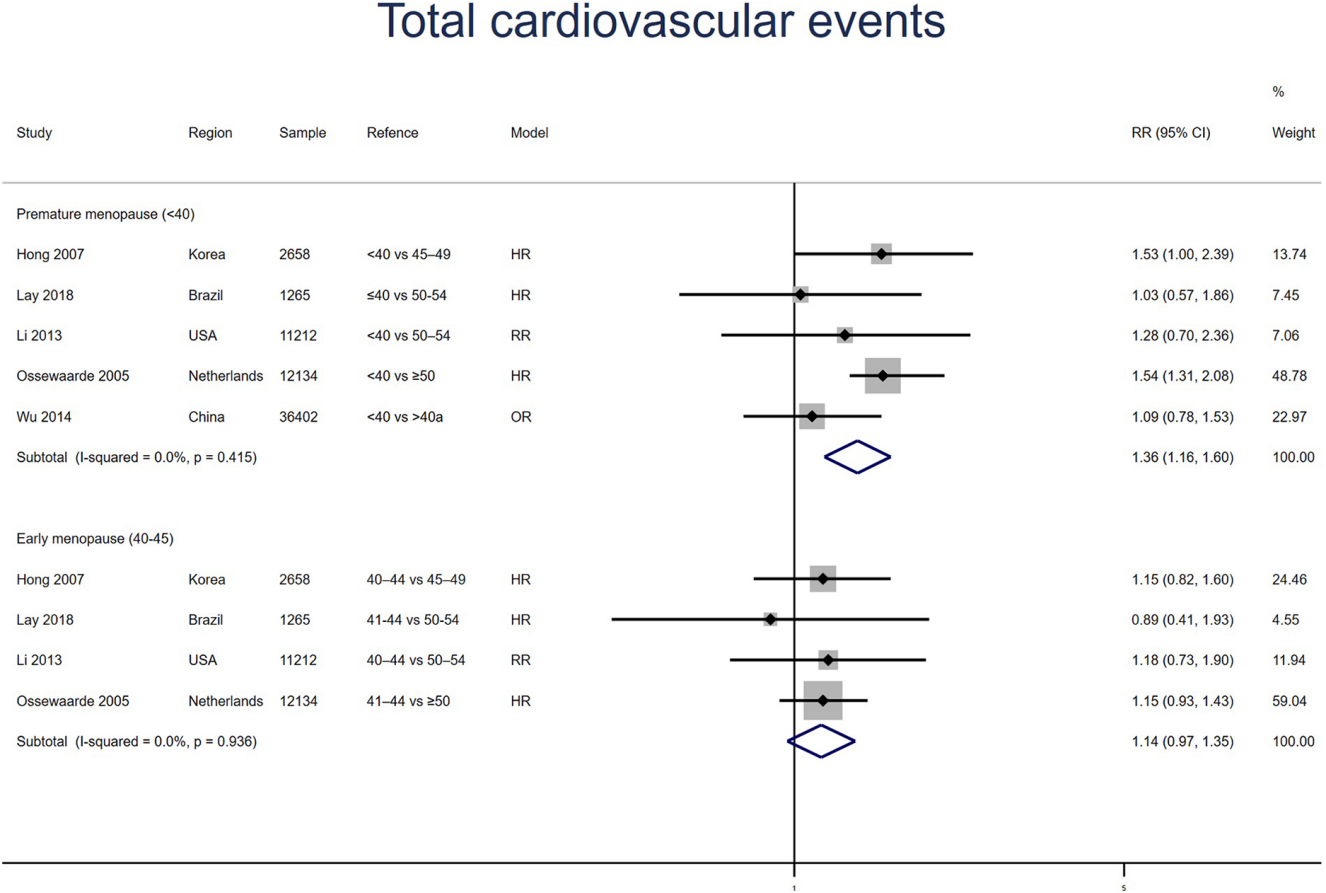
Forest plot presents the association between PM or EM and the risk of fatal cardiovascular events in cohort study.

Five studies for PM and four studies for EM reported total cardiovascular events, with no heterogeneity between the studies (*I*^2^ = 0%). Meta-analysis showed that both PM (pooled RR: 1.36, 95% CI: 1.16–1.60) and EM (pooled RR: 1.14, 95% CI: 0.97–1.35) had an increased risk of total cardiovascular events (Figure 9). No significant publication bias was found in Egger's test (*p* = 0.357 for PM, *p* = 0.333 for EM).

## Discussion

In this meta-analysis, based on 20 cohort studies, we found that premature or early menopausal women had an increased risk of cardiovascular risk factors, such as T2D and HL, but not of HT in comparison to women in the reference category. Both PM and EM were linked to an increased risk of CHD, stroke, as well as total CVD events.

### The results for stroke were inconsistent

In this study, we also found that PM women, but not EM women, had an elevated risk of hemorrhagic and ischemic stroke. This is not in line with the conclusion that both PM and EM women had a higher risk of total stroke. This may be caused by the following reasons: Four studies (20, 24, 31, 34) reported ischemic and hemorrhagic stroke separately and one study (17) reported ischemic stroke, most of them observed a limited number of events (17, 24, 31, 34). Furthermore, due to the late application of modern medical instruments such as computed tomography (CT), only a small number (18.5%) of the previously published cohort were specifically recorded as ischemic or hemorrhagic stroke (24). Finally, four of the included studies (20, 24, 31, 34) did not distinguish the type of stroke in some patients, it may be that hospital medical staff failed to provide specific information on the type of stroke or that the code of the disease was not validated. Therefore, the rate of ischemic or hemorrhagic stroke in the included studies may not have been as high as it should have been, and our findings regarding the associations between ischemic or hemorrhagic stroke and PM or EM may not be as reliable. However, in current systematic reviews, the relationship between PM or EM and the increased risk of stroke was not consistent. A recent meta-analysis of 78 studies reported that an earlier age at menopause is significantly associated with an increased risk of hemorrhagic stroke but not significantly total and ischemic stroke (35). And a meta-analysis included 10 prospective cohorts found total stroke, ischemic stroke, and hemorrhagic stroke were not increased in POI women (9). These results suggest that the relationship between menopausal age and the risk of stroke remains controversial, and further well-designed prospective cohorts with adequate samples are required to clarify this association.

### Mechanisms of CVD in early menopausal women

The higher cardiometabolic risk in earlier menopausal women may involve the following mechanisms: Estrogen exerts a positive protective effect on the cardiovascular system, and a longer reproductive lifespan (representing longer duration of estrogen exposure) is associated with a lower risk of CVD events (36). Early estrogen depletion may exacerbate the expression of inflammatory chemicals and decrease vascular function (37). In a cross-sectional study comparing early vs. late menopause, higher estrogen levels were linked to smaller carotid interadventitic diameter (reflecting less carotid remodeling) and increased flow-mediated dilatation of the brachial artery (suggestive of better endothelial function) (38). Additionally, declining estrogen levels are linked to higher levels of a wide variety of cardiovascular risk factors (39, 40). PM or EM is accompanied by abnormal glucose and lipid metabolism, which are independent and important cardiovascular risk factors. A meta-analysis included 21 studies reported that earlier menopause was linked to significantly higher lipid and glucose levels (41). Also, hyperandrogenism and low sex hormone-binding globulin are involved in the development of CVDs (42).

### The influence of confounders

The cohort studies examined in this analysis adjusted for diverse confounding factors including MHT. There were not enough studies to demonstrate whether the results were independent of MHT, a major confounder in the association between earlier menopause and CVD (16). This issue may be better addressed by a meta-analysis included detailed information on each participant. According to a meta-analysis, the impact of estrogen on CVD was related to the age of menopause and the age at which MHT began. The risk of CHD in women who started MHT soon after menopause was significantly reduced, with a RR of 0.72 (95% CI: 0.56–0.92) when using estrogen plus progestin. The RR of estrogen-progestin administration more than 10 years after menopause was 0.90 (95% CI: 0.62–1.29). The RR of CHD was 1.07 (95% CI: 0.65–1.78) in women older than 60 years who started hormone therapy (43). Observational studies have shown that MHT can provide CVD benefits, and this benefit may be more evident in early menopausal women (26, 44). The International Menopause Society (IMS) published a recommendation on MHT in 2016: starting and long-term use of estrogen has cardiovascular protective effects in perimenopausal and postmenopausal women under the age of 60, and it is not recommended to use MHT to prevent CHD in women over the age of 60 (45). However, for women with established CVD, there is currently no strong evidence that hormones improve cardiac outcomes. The HERS study included 2,763 women with CHD at a mean age of 66.7 years. At a mean follow-up of 4.1 years, the treatment group's relative risk for myocardial infarction or death from CHD was 0.99 (95% CI: 0.80–1.22) compared to the control group, but the treatment group's risk of CHD was higher in the first year. While the risk decreased in 3–5 years, to determine whether the reduction in risk was sustained, a subsequent follow-up of 2.7 years was conducted, and the risk of CHD did not decrease (46). The results suggest that MHT should not be utilized to lower the risk of recurrent CHD events in women with CVD.

The form of menopause was also a confounder. During natural menopause, hormone levels decline gradually, and for many years after menopause, large amounts of testosterone are still produced and peripherally converted to estrogen. With bilateral oophorectomy, hormone levels are abruptly decreased, resulting in acute hypoestrogenic and hypoandrogenic conditions (47). Different types of menopause may lead to inconsistent postmenopausal hormone levels, which are associated with different cardiovascular risks. However, three studies included in this analysis separately analyzed and reported the effects of natural and surgical menopause on cardiovascular outcomes, and no significant associations were observed (17, 20, 34). This is consistent with another cohort study that specifically examined the relationship between natural or surgical menopause and CVD (48). According to this study, the cardiovascular risk profile before surgery already determines the future cardiovascular risk of women undergoing oophorectomy and has little to do with the surgery itself.

### Strengths and limitations

To our knowledge, this is the first systematic assessment that reports on all significant cardiometabolic endpoints based on longitudinal cohort studies. The strength of this study is that the literature included was of high quality, scoring over 7 points according to the NOS. Furthermore, we conducted subgroup analysis to examine the relationship of PM and EM with cardiovascular risk factors and CVD events separately, which may allow for risk stratification based on age at menopause and better patient management. Moreover, sufficient numbers of American, Asian, and European populations were included in our study, which allowed us to perform subgroup analysis according to ethnicity. We used multiple-adjusted effect sizes to avoid the effect of confounders on the final results, unfortunately, the confounders varied across studies. Only analyzing the data for each individual would possibly address this question.

Our study has certain limitations, mainly in terms of clinical heterogeneity. The reference age for normal menopause was not identical between studies, and the outcome measures were affected by differences in the reporting methods, some from self-reports and some from device diagnoses. The clinical heterogeneity of the literature may influence how generalizable our findings are to specific clinical contexts using different reference categories. This is the primary limitation of our study and other studies have examined the association between earlier menopause and the increased risk of CVD. Moderate heterogeneity (*I*^2^ > 50%) existed in the systematic review for T2D, HT, CHD (<40), stroke (40–45), ischemic stroke (40–45). Considerable heterogeneity (*I*^2^ > 70%) existed for stroke (<40), but removing outliers decreased the heterogeneity without changing the results. No heterogeneity was found for HL, CHD (40–45), ischemic stroke (<40), hemorrhagic stroke, or fatal cardiovascular events. Second, the causes of EM and PM, including oophorectomy or spontaneity, may have independent effects on CVD. Unfortunately, most studies only included natural menopausal women or treated surgical menopause as a confounding factor without comparing the cardiovascular risks of natural and surgical menopause. Considering the few included studies separately analyzing natural and surgical menopause, we are not available to perform subgroup analysis based on the pattern of menopause. However, a very recent observational study had shown that, when compared to natural menopause, surgical menopause did not significantly increase the risk of total stroke (34). Third, postmenopausal hormone use may have an impact on the relationship between earlier age at menopause and CVD (49). We could not assess the effect of MHT use, a potential major confounder, on CVD in early or premature menopausal women. However, studies have shown that about 80% of women use hormone treatment for at least 6 years after menopause (50). Therefore, we can assume that the bias due to hormone use is very small (16). Fourth, the self-reported age of menopause might lead to recall bias. However, some articles have reported that the majority of women accurately described the state of menopause, indicating that self-reported menopausal age is reasonably reliable (51). Besides, considering that each article included standard questionnaires and detailed questions, we assume that the effect of self-reported age at menopause on the final outcome is limited.

### Public health value

Our results may have useful public health value. First, compared with smoking, hypertension, and diabetes (OR: 2.86, 95% CI: 2.36–3.48; OR: 2.95, 95% CI: 2.57–3.39; OR: 4.26, 95% CI: 3.51–5.18, respectively) (52), PM or EM is a moderate risk factor for CVD. Although modest, the increased CVD risk is significant. Therefore, maintaining a healthy lifestyle, such as quitting smoking and exercising on a regular basis (53), is the cornerstone of CVD prevention in early menopausal women. Second, the timing of MHT initiation affects its cardiovascular effects, namely the “timing hypothesis” (45). Timely initiating MHT in early menopausal women not only improves symptoms and quality of life but also has cardiovascular benefits that can be used as primary CVD prevention. Third, data for secondary prevention of CVD and improved survival for women are still lacking, with further research needed to develop evidence-based recommendations specifically for women (54).

## Conclusion

In summary, this study included 20 cohorts showed that women with early menopausal age (< 40 years or 40–45 years) had an increased risk of developing CVD. This needs to be taken into consideration when developing prognostic models for the early diagnosis of CVD in women, particularly in those at higher risk, which requires timely lifestyle intervention and possibly hormonal treatment. Prospective cohort studies with larger sample sizes and well-designed intervention studies may help to address this issue.

## Data Availability

The original contributions presented in the study are included in the article/**Supplementary Material**, further inquiries can be directed to the corresponding author.
