# Supplementary material for: The risk of long-term cardiometabolic disease in women with premature or early menopause: A systematic review and meta-analysis
Source: Front Cardiovasc Med. 2023 Mar 21;10:1131251. doi: 10.3389/fcvm.2023.1131251 (PMC10072266; doi:10.3389/fcvm.2023.1131251)
Supplement: Supplementary file 1 [file Table2.docx]

**Supplementary table 1**

PUBMED (Searched on: 1 October 2022)

| Search number | Query | Results | Time |
| --- | --- | --- | --- |
| #1 | "Primary Ovarian Insufficiency"[Mesh] | 3285 | 18:30:03 |
| #2 | ((((((((((Primary Ovarian Failure[Title/Abstract]) OR (Premature Ovarian Failure[Title/Abstract])) OR (Premature Ovarian Insufficiency[Title/Abstract])) OR (early menopause[Title/Abstract])) OR (Hypergonadotropic Ovarian Failure, X Linked[Title/Abstract])) OR (Premature Ovarian Failure, X Linked[Title/Abstract])) OR (Fragile X Associated Primary Ovarian Insufficiency[Title/Abstract])) OR (Fragile X Premature Ovarian Failure[Title/Abstract])) OR (FMR1 Related Primary Ovarian Insufficiency[Title/Abstract])) OR (Primary Ovarian Insufficiency, Fragile X Associated[Title/Abstract])) OR (Gonadotropin Resistant Ovary Syndrome[Title/Abstract]) | 5044 | 18:32:22 |
| #3 | #1OR#2 | 6173 | 18:33:01 |
| #4 | ((("Hypertension"[Mesh]) OR ("Cardiovascular Diseases"[Mesh])) OR ("Diabetes Mellitus"[Mesh])) OR ("Lipids"[Mesh]) | 4,102,655 | 18:35:17 |
| #5 | ((((((((((((((((((((((((((((((((((((((Hypertension[Title/Abstract]) OR (High Blood Pressure[Title/Abstract])) OR (Blood Pressure, High[Title/Abstract])) OR (Cardiovascular Disease[Title/Abstract])) OR (Cardiovascular Risk[Title/Abstract])) OR (Cardiometabolic Risk[Title/Abstract])) OR (Disease, Cardiovascular[Title/Abstract])) OR (Cardiovascular Symptom[Title/Abstract])) OR (Cardiovascular Mortality[Title/Abstract])) OR (Brain Ischemia[Title/Abstract])) OR (Heart Muscle Ischemia[Title/Abstract])) OR (Silent Myocardial Ischemia[Title/Abstract])) OR (Ischemic Heart Disease[Title/Abstract])) OR (Coronary Artery Disease[Title/Abstract])) OR (Myocardial Disease[Title/Abstract])) OR (Cerebrovascular Disease[Title/Abstract])) OR (Carotid artery Disease[Title/Abstract])) OR (Cerebral artery Disease[Title/Abstract])) OR (Cerebrovascular Accident[Title/Abstract])) OR (Stroke[Title/Abstract])) OR (Strokes[Title/Abstract])) OR (Heart Diseas*[Title/Abstract])) OR (CVD[Title/Abstract])) OR (CHD[Title/Abstract])) OR (IHD[Title/Abstract])) OR (CVA[Title/Abstract])) OR (Diabetes Mellitus[Title/Abstract])) OR (DM[Title/Abstract])) OR (Lipids[Title/Abstract])) OR (Hyperlipidemias[Title/Abstract])) OR (Cholesterol[Title/Abstract])) OR (Triglycerides[Title/Abstract])) OR (Cholesterol[Title/Abstract])) OR (Cholesterol, LDL[Title/Abstract])) OR (Cholesterol, HDL[Title/Abstract])) OR (LDL-C[Title/Abstract])) OR (HDL-C[Title/Abstract])) OR (TC[Title/Abstract])) OR (TG[Title/Abstract]) | 1,782,536 | 18:43:57 |
| #6 | #4OR#5 | 4,713,097 | 18:46:18 |
| #7 | #3AND#6 | 774 | 18:51:08 |

EMBASE (Searched on: 1 October 2022)

| Search number | Query | Results | Time |
| --- | --- | --- | --- |
| #1 | 'premature ovarian failure'/exp | 6003 | 19:12:11 |
| #2 | 'primary ovarian insufficiency':ti,ab OR 'primary ovarian failure':ti,ab OR 'premature ovarian failure':ti,ab OR 'premature ovarian insufficiency':ti,ab OR 'early menopause':ti,ab OR 'premature ovarian failure, x linked':ti,ab OR 'hypergonadotropic ovarian failure, x linked':ti,ab OR 'fragile x associated primary ovarian insufficiency':ti,ab OR 'fragile x premature ovarian failure':ti,ab OR 'fmr1 related primary ovarian insufficiency':ti,ab OR 'primary ovarian insufficiency, fragile x associated':ti,ab OR 'gonadotropin resistant ovary syndrome':ti,ab | 8433 | 19:17:23 |
| #3 | #1OR#2 | 9830 | 19:18:13 |
| #4 | 'hypertension'/exp OR 'cardiovascular disease'/exp OR 'diabetes mellitus'/exp OR 'lipid'/exp | 7137965 | 19:22:09 |
| #5 | 'hypertension':ab,ti OR 'high blood pressure':ab,ti OR 'blood pressure, high':ab,ti OR 'cardiovascular disease':ab,ti OR 'cardiovascular risk':ab,ti OR 'cardiometabolic risk':ab,ti OR 'disease, cardiovascular':ab,ti OR 'cardiovascular symptom':ab,ti OR 'cardiovascular mortality':ab,ti OR 'brain ischemia':ab,ti OR 'heart muscle ischemia':ab,ti OR 'silent myocardial ischemia':ab,ti OR 'ischemic heart disease':ab,ti OR 'coronary artery disease':ab,ti OR 'myocardial disease':ab,ti OR 'cerebrovascular disease':ab,ti OR 'carotid artery disease':ab,ti OR 'cerebral artery disease':ab,ti OR 'cerebrovascular accident':ab,ti OR 'stroke':ab,ti OR 'strokes':ab,ti OR 'heart diseas*':ab,ti OR 'cvd':ab,ti OR 'chd':ab,ti OR 'ihd':ab,ti OR 'cva':ab,ti OR 'diabetes mellitus':ab,ti OR 'dm':ab,ti OR 'lipids':ab,ti OR 'hyperlipidemias':ab,ti OR 'triglycerides':ab,ti OR 'cholesterol':ab,ti OR 'cholesterol, ldl':ab,ti OR 'cholesterol, hdl':ab,ti OR 'ldl-c':ab,ti OR 'hdl-c':ab,ti OR 'tc':ab,ti OR 'tg':ab,ti | 2494356 | 19:27:46 |
| #6 | #4OR#5 | 7548157 | 19:28:07 |
| #7 | #3AND#6 | 1841 | 19:33:52 |

WOS (Searched on: 1 October 2022)

| Search number | Query | Results | Time |
| --- | --- | --- | --- |
| #1 | (((((((((((TS=(Primary Ovarian Insufficiency)) OR TS=(Primary Ovarian Failure)) OR TS=(Premature Ovarian Failure)) OR TS=(Premature Ovarian Insufficiency)) OR TS=(early menopause)) OR TS=(Hypergonadotropic Ovarian Failure, X Linked)) OR TS=(Premature Ovarian Failure, X Linked)) OR TS=(Fragile X Associated Primary Ovarian Insufficiency)) OR TS=(Fragile X Premature Ovarian Failure)) OR TS=(FMR1 Related Primary Ovarian Insufficiency)) OR TS=(Primary Ovarian Insufficiency, Fragile X Associated)) OR TS=(Gonadotropin Resistant Ovary Syndrome) | 10,647 | 20:32:07 |
| #2 | ((((((((((((((((((((((((((((((((((((((TS=(Hypertension)) OR TS=(High Blood Pressure)) OR TS=(Blood Pressure, High)) OR TS=(Cardiovascular Disease)) OR TS=(Cardiovascular Risk)) OR TS=(Cardiometabolic Risk)) OR TS=(Disease, Cardiovascular)) OR TS=(Cardiovascular Symptom)) OR TS=(Cardiovascular Mortality)) OR TS=(Brain Ischemia)) OR TS=(Heart Muscle Ischemia)) OR TS=(Silent Myocardial Ischemia)) OR TS=(Ischemic Heart Disease)) OR TS=(Coronary Artery Disease)) OR TS=(Myocardial Disease)) OR TS=(Cerebrovascular Disease)) OR TS=(Carotid artery Disease)) OR TS=(Cerebral artery Disease)) OR TS=(Cerebrovascular Accident)) OR TS=(Stroke)) OR TS=(Strokes)) OR TS=(Heart Diseas*)) OR TS=(CVD)) OR TS=(CHD)) OR TS=(IHD)) OR TS=(CVA)) OR TS=(Diabetes Mellitus)) OR TS=(DM)) OR TS=(Lipids)) OR TS=(Hyperlipidemias)) OR TS=(Cholesterol)) OR TS=(Triglycerides)) OR TS=(Cholesterol)) OR TS=(Cholesterol, LDL)) OR TS=(Cholesterol, HDL)) OR TS=(LDL-C)) OR TS=(HDL-C)) OR TS=(TC)) OR TS=(TG) | 2,621,424 | 20:38:42 |
| #3 | #1AND#2 | 1,824 | 20:50:37 |
