# Supplementary material for: The risk of long-term cardiometabolic disease in women with premature or early menopause: A systematic review and meta-analysis
Source: Front Cardiovasc Med. 2023 Mar 21;10:1131251. doi: 10.3389/fcvm.2023.1131251 (PMC10072266; doi:10.3389/fcvm.2023.1131251)
Supplement: Supplementary file 2 [file Table3.docx]

| Studies | Representativeness of the exposed cohort | Selection of non-exposed cohort | Ascertainment of exposure factor | Demonstration that outcome of interest was not present at start of study | Comparability of cohorts on the basis of the design or analysis (★★) | Evaluation of outcome | Was follow-up long enough for outcomes to occur | Adequacy of follow-up of cohorts | Quality Scores |
| --- | --- | --- | --- | --- | --- | --- | --- | --- | --- |
| Baba 2010 | × | ★ | ★ | ★ | ★★ | ★ | ★ | × | 7 |
| Blümel 2022 | ★ | ★ | ★ | ★ | ★★ | ★ | ★ | × | 8 |
| Brand 2013 | ★ | × | ★ | ★ | ★★ | ★ | ★ | × | 7 |
| Choi 2005 | × | ★ | ★ | × | ★★ | ★ | ★ | ★ | 7 |
| Cooper 1998 | × | ★ | ★ | - | ★★ | ★ | - | ★ | 6 |
| Gallagher 2011 | × | ★ | × | ★ | ★★ | ★ | ★ | ★ | 7 |
| Hong 2007 | ★ | ★ | ★ | ★ | ★★ | ★ | ★ | ★ | 9 |
| Hu 1999 | × | ★ | ★ | ★ | ★★ | ★ | ★ | ★ | 8 |
| Jacobsen 1999 | × | ★ | ★ | ★ | ★★ | × | ★ | ★ | 8 |
| Jacobsen 2004 | × | ★ | ★ | ★ | ★★ | ★ | ★ | ★ | 8 |
| Lay 2018 | ★ | ★ | ★ | - | ★★ | ★ | ★ | ★ | 8 |
| Li 2013 | ★ | ★ | ★ | ★ | ★★ | ★ | ★ | × | 8 |
| Løkkegaard 2006 | × | ★ | ★ | ★ | ★★ | ★ | ★ | ★ | 8 |
| Mondul 2005 | ★ | × | ★ | ★ | ★★ | ★ | ★ | × | 8 |
| Muka 2017 | ★ | ★ | ★ | ★ | ★★ | ★ | ★ | ★ | 9 |
| Ossewaarde 2005 | ★ | ★ | ★ | × | ★★ | ★ | ★ | ★ | 8 |
| Wang 2022 | ★ | ★ | ★ | ★ | ★★ | ★ | ★ | ★ | 9 |
| Welten 2021 | ★ | × | ★ | ★ | ★★ | ★ | ★ | ★ | 8 |
| Wu 2014 | ★ | ★ | ★ | ★ | ★★ | ★ | ★ | × | 8 |
| Zhu 2019 | ★ | ★ | ★ | ★ | ★★ | ★ | ★ | ★ | 9 |

Notes: “★” represents 1 point, “×” represents 0 point, and “—” represents uncertain points.

Abbreviation: NOS, Newcastle–Ottawa Scale.
